# Coencapsulation of Multistrain *Bacillus* Spores With Alginate and *Gracilaria* Polysaccharide Enhances Stability, Egg Production, and Gut Health in Laying Hens

**DOI:** 10.1155/ijm/8530924

**Published:** 2026-05-06

**Authors:** Waraphorn Sihamok, Orathai Dangsawat, Apinan Nuisiri, Jessada Rattanawut, Rapeewan Sowanpreecha, Umaporn Pastsart, Chatchawan Chotimarkorn, Papungkorn Sangsawad, Luu Tang Phuc Khang, Orranee Srinual, Nguyen Vu Linh, Patima Permpoonpattana

**Affiliations:** ^1^ Department of Agricultural Science and Technology, Prince of Songkla University, Surat Thani Campus, Surat Thani, Thailand, psu.ac.th; ^2^ Scientific Laboratory and Equipment Center, Prince of Songkla University, Surat Thani Campus, Surat Thani, Thailand, psu.ac.th; ^3^ Department of Food Technology, Prince of Songkla University, Surat Thani Campus, Surat Thani, Thailand, psu.ac.th; ^4^ School of Animal Technology and Innovation, Suranaree University of Technology, Nakhon Ratchasima, Thailand, sut.ac.th; ^5^ Department of Animal and Aquatic Sciences, Chiang Mai University, Chiang Mai, Thailand, cmu.ac.th

**Keywords:** *Bacillus*, coencapsulation, laying hens, poultry, sodium alginate

## Abstract

Stable and effective probiotic supplements are essential for sustainability in animal production. To optimize delivery systems and evaluate the in vivo performance of a multistrain spore‐forming probiotic for laying hens, a comparative study was conducted. Sodium alginate alone (AO) and an alginate–*Gracilaria fisheri* polysaccharide compound (AP) were evaluated as encapsulation matrices. This study aimed to assess the efficacy and safety of a multistrain probiotic supplement for laying hens. The supplement was formulated with spores from four promising *Bacillus* strains including *Bacillus aryabhattai* CKNJH11, *Bacillus* sp. THPS1, *Lysinibacillus* sp. PWR01, and *Bacillus marisflavi* OYNH19. The results showed that coencapsulating spores with AP demonstrated a significantly higher encapsulation yield (*p* < 0.05) and improved survival under simulated gastrointestinal conditions, heat treatment, lysozyme exposure, and 60‐day storage. In vivo feeding trials with Hi‐sex Brown laying hens (35 weeks) showed that AP microcapsules delivering 1 × 10^6^ spores/g feed significantly increased egg production (peak at 95.88%), body‐weight gain, and egg quality compared to the control and free‐spore groups (*p* < 0.05). Feed conversion ratio (FCR) and Haugh unit (HU) remained unchanged (*p* > 0.05) at 38 weeks. Additionally, hens fed the encapsulated probiotic spores demonstrated a notably decreased fecal *Salmonella* sp. level (5.29–5.40 log_10_ CFU/g) when compared to control groups (*p* < 0.05). Coencapsulating multistrain *Bacillus* spores with AP improved spore stability and maintained measurable production and gut‐health–related outcomes under laying hen conditions, supporting encapsulation‐matrix selection as a determinant of probiotic product performance in poultry production.

**Highlights**
• Alginate + polysaccharide coencapsulation stabilizes *Bacillus* spores.
• Coencapsulation feed improves egg production and egg quality in laying hens.
• Coencapsulation feed reduces fecal *Salmonella* sp. level (5.29–5.40 log_10_ CFU/g).

## 1. Introduction

Overuse of antibiotics in animal feed has raised significant concerns about antibiotic‐resistant microorganisms and food residue accumulation [1]. Antibiotics are extensively utilized in animals both therapeutically and as growth promoters to augment body‐weight gain (BWG) and prevent infections [2, 3]. This practice has resulted in the emergence of antibiotic‐resistant bacteria that can be transmitted from animals to humans through the food chains [4, 5]. Researchers have identified promising alternatives to antibiotics (e.g., probiotics, prebiotics, essential oils, bacteriophages, antimicrobial peptides, enzymes, or organic acids), which can enhance growth performance, immune response, and disease resistances while simultaneously maintaining animal health [6, 7]. Probiotics, particularly *Bacillus* species, have demonstrated promising sustainable alternatives to antibiotic growth promoters in livestock and aquatic species. *Bacillus* species offer unique advantages due to their spore‐forming capability, allowing them to withstand in harsh conditions such as high temperatures during feed processing, acidic stomach environment, and bile salt in the intestine [8, 9]. These spores demonstrate remarkable resilience with up to 100% survivability in gastrointestinal tract (GIT) conditions and retain approximately 90% viability during harvesting processes [10]. In the poultry industry, *Bacillus* probiotics specially enhance feed utilization and stress response, while reducing pathogenic bacterial loads. These probiotics produce antimicrobial compounds and compete with pathogens for nutrients, thereby strengthening gut barriers and modulating the composition of the microbiomes [11, 12]. Furthermore, they also improve feed efficiency up to 5% and reduce mortality, making them suitable for commercial animal nutrition [10]. Nevertheless, their efficacy exhibits substantial variability and is influenced by multiple critical factors, including the selection of an appropriate strain, dosage, delivery methods, and storage/application conditions [13].

The development of multistrain probiotics for application in laying hens requires systematic strain‐level selection to achieve functional complementarity, processing stability, and production applicability. In this context, four spore‐forming Bacillaceae strains previously isolated from diverse ecological niches in Thailand have been incorporated based on validated probiotic‐relevant properties and documented performance data in poultry. Among the selected strains, *Bacillus aryabhattai* CKNJH11 demonstrates for its high sporulation efficiency, sustained viability under simulated gastric acid and bile salt conditions, nonhemolytic phenotype, and beneficial effects on BWG and eggshell weight (ESW) in laying hens, supporting its role in enhancing gastrointestinal persistence and egg quality [14]. Originating from a thermophilic hot spring habitat, *Bacillus* sp. THPS1 possesses genomic determinants associated with stress resistance, sporulation potential, and biosynthesis of secondary metabolites [15]. Its inclusion is further justified by demonstrated improvement in feed conversion efficiency without compromising physiological stability [16]. Both *Lysinibacillus* sp. PWR01 and *Bacillus marisflavi* OYNH19 exhibited high sporulation and encapsulation efficiencies, contributing robustness to spore‐based delivery systems and may regulate the spatiotemporal dynamics of release within the intestinal tract [16]. Together, these strains represent a functionally complementary consortium integrating gastrointestinal resilience, metabolic optimization, environmental stress tolerance, and formulation stability, thereby providing a mechanistically grounded multistrain platform suitable for evaluation in laying hen production.

Probiotic encapsulation has emerged as a critical technology to address viability challenges during processing, storage, and gastrointestinal transit [17]. Encapsulation creates a physical barrier between microorganisms and external environments, protecting cells during processing, shelf life, and passage through the GIT [18]. Alginate‐based systems are widely utilized due to their biocompatibility, biodegradability, and gel‐forming capabilities through ionic gelation techniques [19]. However, single alginate has limitations in long‐term stability and protection efficiency [20]. Recent studies have highlighted that single‐material encapsulation systems often fail to provide sufficient long‐term stability or consistent probiotic release under the complex and dynamic conditions [13], which could be encountered in poultry production systems, particularly in laying hens where prolonged feeding cycles are required. However, these materials are either relatively costly, derived from nonrenewable sources, or lack additional biological functionality, limiting their scalability and attractiveness for routine use in commercial poultry feeds. In this context, *Gracilaria fisheri*–derived polysaccharide (GFP) represents a highly promising yet underexplored encapsulation material. GFP is a sulfated seaweed polysaccharide abundantly available in Southeast Asian coastal regions, including Thailand, and is characterized by high gel‐forming capacity, hydrophilicity, and strong interaction potential with divalent cations and alginate chains [21, 22]. These properties are expected to complement alginate by reinforcing the gel matrix, reducing porosity, and improving resistance to thermal, acidic, and enzymatic degradation. In addition, seaweed‐derived polysaccharides have been increasingly recognized for their prebiotic potential, biocompatibility, and sustainability, offering both functional and environmental advantages over conventional encapsulation materials [23, 24]. Despite these advantages, the application of GFP in probiotic encapsulation for poultry, particularly laying hens, remains largely unexplored, representing a critical knowledge gap. On the other hand, the urgency of developing more effective encapsulation systems is further underscored by the practical realities of the poultry industry [25–27], where probiotics must withstand high‐temperature feed processing, extended storage without refrigeration, and prolonged administration periods while maintaining consistent efficacy [28, 29]. Therefore, this study hypothesized that coencapsulating four *Bacillus* spores with alginate and GFP would result in a superior probiotic product exhibiting enhanced stability and efficacy. To investigate this, the study assessed the stability of the encapsulation of multistrain *Bacillus* spores in alginate alone (AO) or a combination of alginate and GFP (AP) in different stress conditions. Additionally, we also evaluate their impact on production performance, egg quality, and gut health in laying hens.

## 2. Materials and Methods

### 2.1. Bacterial Culture, Characterization, and Identification

Bacterial isolates were obtained from our previous studies in four different environmental sources: CKNJh11 isolated from shrimp pond soil [14]; THPS1 isolated from hot spring soil [15]; OYNH19 from chicken manure [30]; and PWR01 isolated from rubber latex serum [30]. Briefly, the isolates were cultured on nutrient agar (NA, HiMedia, India) using the spread plate method and incubated at 37°C for 24 h. The colony morphology was observed, and Gram staining was performed to determine the cell morphology and Gram reaction. Biochemical tests, including motility, catalase, oxidase, indole, and starch hydrolysis, were conducted as reported previously [14].

### 2.2. Extraction of Seaweed (*Gracilaria fisheri*) Polysaccharide

*Gracilaria fisheri* (*G. fisheri*) seaweed was collected from Chaiya district, Surat Thani province, Thailand, for the extraction of its polysaccharides. The seaweed was thoroughly washed, sun‐dried, and oven‐dried at 60°C. Twenty grams of dried seaweed was then soaked in a 6% NaOH solution for 5 h at room temperature, followed by incubation in a water bath for 1.5 h. After washing, the seaweed was immersed in a 0.025% H_2_SO_4_ solution for 2 h. After filtration and washing, the seaweed was cut into 1.0–1.5 cm pieces. These pieces were added to 1500 mL of distilled water, where they were allowed to soak for 15 min at a pH range of 6.2–6.8, and then boiled for 1.5 h with intermittent stirring. The extract was filtered through two layers of white cloth, cooled to room temperature, frozen at −20°C for 24 h, thawed under running water, and oven‐dried at 60°C until constant weight was achieved. The dried polysaccharide was ground into fine powder and stored for encapsulation experiments [31].

### 2.3. Probiotic Encapsulation

#### 2.3.1. Production and Preparation of Probiotic Spores

Each *Bacillus* strain was first cultured in nutrient broth (NB) at 37°C for 18–24 h to obtain actively growing vegetative cells. Sporulation was then induced by transferring cultures (1%, v/v) into Difco sporulation medium (DSM) and incubating at 37°C for 48 h under aerobic conditions with gentle agitation (200 rpm) [32]. Sporulation efficiency was monitored microscopically using phase‐contrast microscopy, and cultures exhibiting > 95% refractile spores were harvested. To eliminate residual vegetative cells, cultures were subjected to heat‐shock treatment at 80°C for 20 min [32]. Spore suspensions were centrifuged at 8000 × g for 10 min at 4°C, washed twice with sterile phosphate‐buffered saline (PBS; pH 7.2), and resuspended in sterile distilled water. Equal volumes of spores from each isolate were then combined to obtain a multistrain spore mixture.

#### 2.3.2. Encapsulation Procedure

Spores from all four isolates were mixed in 1 × 10^6^ spores/g prior to encapsulation and encapsulated using two methods: (1) 2% (w/v) sodium alginate alone (AO) and (2) 2% (w/v) sodium alginate combined with 0.8% (w/v) polysaccharide derived from *G. fisheri* (AP). The spore–alginate mixture was dispensed into a 0.45 M CaCl_2_ solution using a syringe and allowed to solidify for 45 min. The beads were filtered, washed with sterile distilled water, and dried at 50°C–70°C. The diameter of the beads was measured using a Vernier caliper (*n* = 10).

#### 2.3.3. Measurement of Encapsulation Efficiency

Encapsulation efficiency was quantified by comparing the viable spore count in beads to the initial spore concentration. Briefly, 1 g of beads was subjected to homogenization in 9 mL of phosphate buffer (pH 7.4). Subsequently, the mixture was centrifuged at 8000 rpm for 10 min at 4°C. The supernatant was then serially diluted and plated onto NA agar. The encapsulation efficiency (EE) was calculated as

(1) EE %=number of spores in beads after encapsulation CFU/g initial number of spores in beads before encapsulation CFU/g×100.

#### 2.3.4. Measurement of Spore Release Efficiency

One gram of encapsulated spores was placed in a sterile glass bottle and dissolved in 9 mL of phosphate buffer (concentration). The mixture was incubated at room temperature for 2 h and then diluted 10‐fold with 0.85% NaCl. The number of released spores was then counted by spread plate method on NA with 3 replicates per sample. The plates were incubated at 37°C for 24 h, and the percentage of spore release (SPR) was calculated as follows:

(2) SPR %=number of released spores after encapsulation CFU/g initial number of released spores before encapsulation CFU/g×100.

#### 2.3.5. Acidity, Bile Salts, Heat, and Lysozyme Resistance

In the context of acidity and bile salt resistance, the tests were conducted in accordance with the previously reported protocols [14, 15] to assess its resistance to pH at concentrations of 2.0, 2.5, and 3.0 and bile salts at concentrations of 0.1%, 0.3%, and 0.5%.

For heat treatment, the encapsulated spores were subjected to a control bath. The survival rate (SR) was calculated by diluting it 10 times with 0.85% NaCl, spreading it on NA agar, curing at 37°C for 24 h, and calculating the SR according to the formula as follows:

(3) SR %=number of spores after heating CFU/g initial number of spore before heating CFU/g×100.

For lysozyme resistance, the tests were conducted in accordance with the protocols previously reported [14, 15]. Briefly, the encapsulated spores were mixed with lysozyme in a ratio of 1:1 (v/v). The mixture was then incubated at 37°C for 30, 60, and 120 min. The SR of the spores was calculated according to the previous report [14].

#### 2.3.6. Storage Testing

The encapsulated spores were stored at temperatures of 4, 25, 37, and 45°C for a period of 60 days. The results were collected every 7 days. The viability of bacterial spores was checked, and the SR of the spores was calculated [14].

#### 2.3.7. Spore Viability Testing

Encapsulated spores were mixed with sterilized chicken feed (TM Feed Products Co., Ltd., Nakorn Pathom, Thailand) at a ratio of 1 g gel beads per 100 g feed, corresponding to a final dose of approximately 1 × 10^6^ viable spores/hen/day and air‐dried at room temperature. The samples were then stored at temperatures of 4, 25, 37, and 45°C for 7, 18, and 60 days. The experiment was conducted with 3 replicates. Subsequently, 1 g of chicken feed was diluted with 0.85% NaCl, spread on NA agar plates, and incubated at 37°C for 24 h to assess the viability of encapsulated spores [14].

### 2.4. In Vivo Feeding Trial

#### 2.4.1. Ethic Statement

The animal experiment protocol was reviewed and approved by the Institutional Animal Care and Use Committee (IACUC) of the Prince of Songkla University (Approval No. AG148/2024). All animal‐related procedures were executed in accordance with the university’s established protocols for the care and utilization of experimental animals.

#### 2.4.2. Experimental Animals, Housing, and Management

**FIGURE 1:**
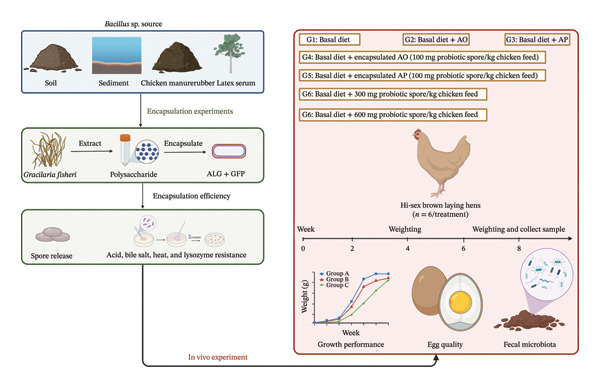
Schematic diagram for experimental process in this study. G1: basal diet (control); G2: basal diet + empty alginate (AO) beads; G3: basal diet + empty AP beads; G4: basal diet + AO‐encapsulated spores (100 mg probiotic spore/kg chicken feed); G5: basal diet + AP‐encapsulated spores (100 mg probiotic spore/kg chicken feed); G6: basal diet + free probiotic spores (300 mg probiotic spore/kg chicken feed); G7: basal diet + free probiotic spores (600 mg probiotic spore/kg chicken feed).

The experiment was conducted using Hi‐sex Brown laying hens (35 weeks of age). The experimental design employed a completely randomized design (CRD), dividing the 42 hens into seven groups, with six hens in each group (Figure 1). The basal diet formulation utilized in the current study was derived from our previous research study [14]. The experiment lasted for 8 weeks. The experimental groups were as follows:

• G1: basal diet (control).
• G2: basal diet + empty AO beads.
• G3: basal diet + empty AP beads.
• G4: basal diet + AO‐encapsulated spores (100 mg probiotic spore/kg chicken feed).
• G5: basal diet + AP‐encapsulated spores (100 mg probiotic spore/kg chicken feed).
• G6: basal diet + free probiotic spores (300 mg probiotic spore/kg chicken feed).
• G7: basal diet + free probiotic spores (600 mg probiotic spore/kg chicken feed).

#### 2.4.3. Egg Quality and Production Performance Analysis

To identify the effect of dietary supplementation on egg quality, including eggshell thickness (EST), shell strength, shell percentage, egg yolk weight (YW), egg white weight, egg white freshness, and egg yolk color, three hen per treatments were collected and subjected to analysis. Additionally, the amount of feed consumed and the feed conversion of ratio were recorded weekly. The measurement was calculated as follows:

(4) feed intake gday=feed given−remaining feed,

(5) body weight gain BWG,g=final body weight−initial body weight,

(6) feed conversion ratio FCR=total feed consumedtotal egg weight,

(7) egg production %=number of eggs collectedtotal number of hens×100,

(8) egg mass g/henday=number of eggs per day×average weight of eggs per replicatenumber of hens per replicate.

#### 2.4.4. Fecal Microbiota Analysis

Fresh fecal samples (*n* = 3) from hens in each treated group were collected at the end of the feeding trial. Briefly, 1 g of feces was homogenized in 9 mL of 0.85% sodium chloride, serially diluted, and plated on selective media including xylose lysine deoxycholate agar for *Salmonella* sp., eosin–methylene blue agar for *Escherichia coli*, and De Man–Rogosa–Sharpe agar for lactic acid bacteria. The plates were incubated at 37°C for 24–48 h, and the colonies were counted.

### 2.5. Statistical Analysis

Data normality was verified using the Shapiro–Wilk test to ensure the assumptions of parametric analysis. One‐way analysis of variance (ANOVA) was applied to assess differences among dietary treatments, and significant effects were further examined using Tukey’s multiple range test for pairwise comparisons. All statistical analyses were performed using IBM SPSS Statistics, version 29.0.2.0 (IBM Corp., Armonk, NY, USA), with statistical significance defined at *p* < 0.05. Results are expressed as mean ± standard error of the mean (SEM). Data visualization was conducted using OriginPro, version 2021b (OriginLab Corporation, Northampton, MA, USA).

## 3. Results

### 3.1. Properties of Encapsulated Spores

Granule diameter was significantly affected by both encapsulation method and drying treatment (Table 1). Wet granules in both AO and AP groups did not differ significantly, but they were larger than their corresponding dry granules (*p* < 0.05). In addition, the cell release rate differed significantly between encapsulation methods, with lower release from alginate‐only granules compared to alginate–polysaccharide granules (*p* < 0.05).

**TABLE 1:** Properties of encapsulated gel granules.

Parameters	Encapsulation methods			
	Alginate only (10^6^ CFU/g)		Alginate and polysaccharide (10^6^ CFU/g)	
	Wet	Dry	Wet	Dry
Diameter (mm)	3.41 ± 0.36^a^	2.89 ± 0.26^b^	3.62 ± 0.35^a^	2.93 ± 0.11^b^
Cell release rate from gel granules (%)	78.78 ± 1.67^b^		98.70 ± 1.12^a^	

*Note:* Data presented as mean ± standard error. Different letters indicated statical significance differences between treatment with *p* < 0.05.

### 3.2. Stability and Tolerance of Encapsulated Spores

The encapsulation method significantly affected spore survival under acidic, bile salt, enzymatic, and heat stress (Figure 2). Under acidic conditions, the AO group exhibited an SR of 89.12%, 90.40%, and 96.89% at pH levels of 2.0, 2.5, and 3.0, respectively, whereas the AP group maintained higher viabilities of 94.50%, 95.68%, and 98.16% (*p* < 0.05) (Figure 2(a)). In bile salt tolerance tests, SR in the AO group decreased slightly with increasing concentration, from 95.59% to 93.96%, while the AP group remained higher, ranging from 96.28% to 97.41% (*p* < 0.05) (Figure 2(b)). For lysozyme resistance, survival declined with increasing exposure time. The AO group decreased from 94.93% at 30 min to 87.61% at 120 min, whereas the AP group retained 97.75%, 93.30%, and 90.44%, respectively (*p* < 0.05) (Figure 2(c)). Heat resistance also differed significantly between treatments. The AO group showed the highest survival at 70°C (95.40%) but declined at 95°C (89.12%), while the AP group maintained high survival at 80°C (96.27%) and 95°C (94.43%) (*p* < 0.05) (Figure 2(d)).

FIGURE 2Stress tolerance of encapsulated spores. (a) Acid tolerance at different pH levels (2, 3, and 4); (b) bile salt tolerance at concentrations of 1, 2.5, and 5%; (c) lysozyme resistance after 30, 60, and 120 min of exposure; and (d) heat resistance at temperatures of 70, 80, and 95°C. Data are presented as the mean ± SEM. Different letters indicate statistically significant differences (*p* < 0.05). AO: alginate‐encapsulated spores; AP: alginate + polysaccharide‐encapsulated spores.

**Figure:**
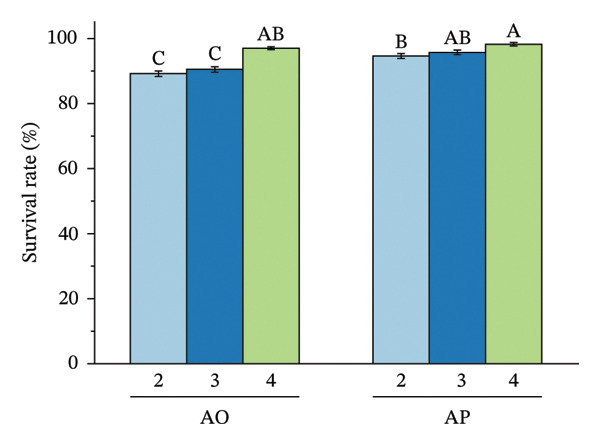
(a)

**Figure:**
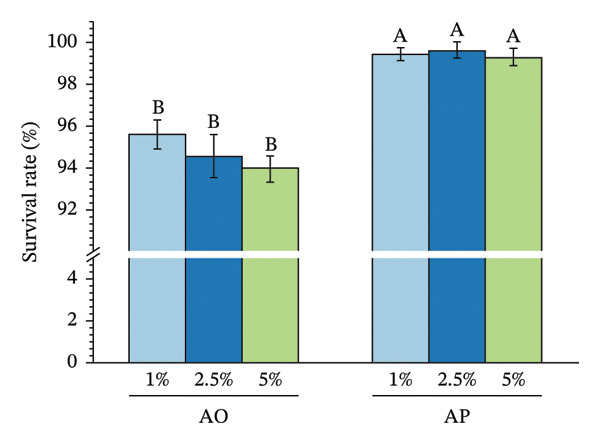
(b)

**Figure:**
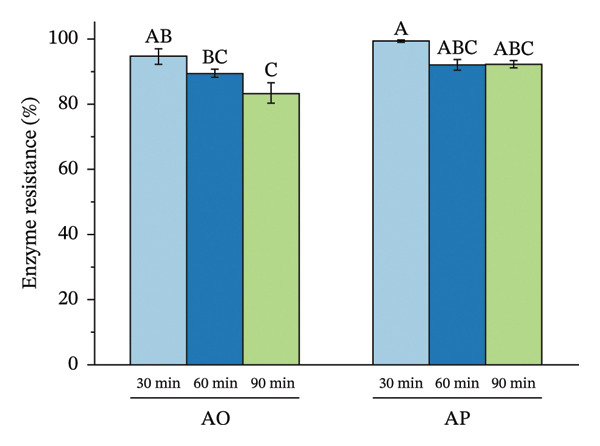
(c)

**Figure:**
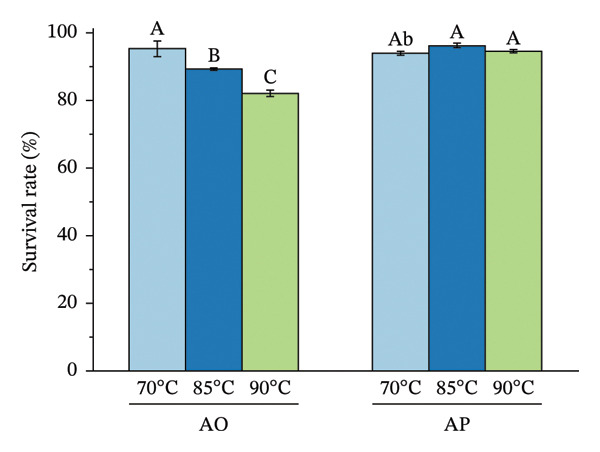
(d)

Spore viability decreased over time and with higher temperatures, with differences observed between encapsulation treatments (Figure 3). At 4°C, spore viability in AO decreased from 95.13 at day 7 to 74.96 at day 60, with significant reductions observed after day 14 (*p* < 0.05). In AP, survival remained 98.37 at day 7 and 97.64 at day 14 and then declined to 84.81 at day 60 (*p* < 0.05). At each time point from day 7 to day 60, AP values were significantly higher than AO (*p* < 0.05).

FIGURE 3Effects of storage temperature and duration on survival (%) of alginate‐coated (AO) and alginate–*Gracilaria fisheri* polysaccharide composite‐coated (AP) probiotic spores at 4°C (a), 25°C (b), and 37°C (c). The minor X axis indicates storage time (days: 7, 14, 21, 30, and 60). Data are presented as the mean ± SEM. Different letters indicate statistically significant differences (*p* < 0.05).

**Figure:**
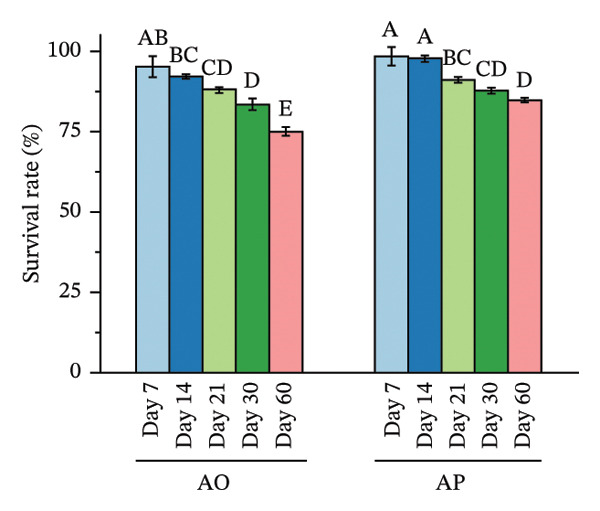
(a)

**Figure:**
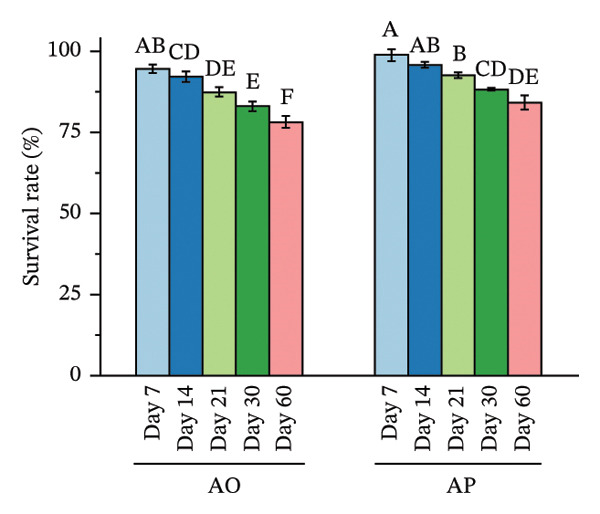
(b)

**Figure:**
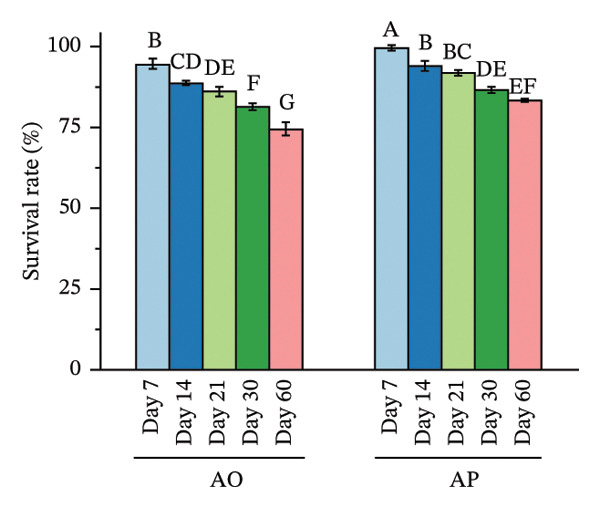
(c)

At 25°C, AO survival declined from 94.54% at day 7% to 78.12% at day 60, with progressive significant decreases after day 14 (*p* < 0.05). In AP, survival decreased from 98.90% at day 7% to 84.20% at day 60 (*p* < 0.05). AP exhibited significantly higher survival than AO at all corresponding time points (*p* < 0.05). At 37°C, AO survival declined from 94.74 at day 7% to 74.61% at day 60, with significant reductions detected after day 7 (*p* < 0.05). In AP, survival decreased from 99.60% at day 7% to 83.32% at day 60 (*p* < 0.05). At each sampling time, AP survival was significantly higher than AO (*p* < 0.05).

### 3.3. Effects on Laying Hen Performance

Probiotic supplementation significantly affected BWG, egg production, and feed intake (Table 2). Weight gain ranged from 10.87 g in G3 to 16.95 g in G5, with G4–G6 showing higher values than G1–G3 (*p* < 0.05). Egg production varied from 90.06% in G3 to 95.88% in G5, with significantly higher percentages observed in G4–G6 compared with those in G1–G3 (*p* < 0.05). Egg mass did not differ significantly among treatments, ranging from 52.83 to 57.64 g/hen/d (*p* > 0.05). Feed intake increased with supplementation, from 97.58 g/d in G2 to 103.58 g/d in G5 (*p* < 0.05). FCR showed no significant differences among groups, remaining between 1.83 and 1.91 (*p* > 0.05).

**TABLE 2:** Effect of probiotic supplementation on production performance of laying hen week 1: 34 (control), week 39 (treatment).

Treatments	Parameters				
	Weight gain (g)	Egg production (%)	Egg mass (g/hen/d)	Feed intake (g/d)	Feed conversion ratio
G1	11.31 ± 0.50	92.06 ± 0.58	53.08 ± 0.80	97.66 ± 1.03	1.83 ± 2.07
G2	11.12 ± 0.87	91.21 ± 0.59	53.17 ± 0.89	97.58 ± 0.92	1.91 ± 1.05
G3	10.87 ± 0.46	90.06 ± 0.68	52.83 ± 0.70	97.87 ± 0.77	1.88 ± 1.07
G4	15.22 ± 0.64	93.60 ± 0.57	55.05 ± 0.76	101.36 ± 0.88	1.90 ± 2.65
G5	16.95 ± 0.73	95.88 ± 0.67	57.64 ± 0.78	103.58 ± 0.82	1.91 ± 0.05
G6	14.24 ± 0.80	94.19 ± 0.45	54.69 ± 0.75	99.33 ± 0.85	1.89 ± 1.19
G7	14.57 ± 0.43	93.57 ± 0.71	54.78 ± 0.79	100.07 ± 1.14	1.88 ± 2.30
SEM	0.42	4.08	1.06	0.85	0.03
*p* value	< 0.001	< 0.001	0.613	< 0.001	0.92

*Note:* Values are expressed as mean ± SEM. Egg mass is expressed as g/hen/day, and feed intake as g/day. Feed conversion ratio was calculated as feed intake divided by egg mass. Data were analyzed using one‐way ANOVA, and differences were considered statistically significant at *p* < 0.05.

### 3.4. Egg Quality

Probiotic supplementation significantly affected egg weight (EW), ESW, eggshell strength (ESS), EST, and albumen weight (AW), whereas YW and Haugh unit (HU) were not altered (*p* ≤ 0.05) (Table 3). EW ranged from 57.00 g (G1) to 66.34 g (G5), with G4–G5 exceeding other groups (*p* < 0.001). ESW increased to 6.50 g (G4) and 6.81 g (G5) compared with 5.01–5.90 g in the remaining groups (*p* < 0.001). ESS rose to 5.37–5.72 kg/cm^2^ in G4–G5 versus 3.94–4.49 kg/cm^2^ in G1–G3 and 4.15–4.46 kg/cm^2^ in G6–G7 (*p* < 0.001). EST was greater in G4–G5 (0.40 mm) than in other groups (0.36–0.39 mm) (*p* = 0.002). AW peaked at 36.78 g (G5) and 35.67 g (G4), exceeding 31.00–35.44 g in other groups (*p* = 0.001). YW (11.84–13.40 g) and HU (90.14–95.25) did not differ among treatments (*p* = 0.228 and *p* = 0.929, respectively) (Table 3).

**TABLE 3:** Effect of probiotic supplementation on production performance of laying hens (35–38 weeks).

Parameters	Treatment								
	G1	G2	G3	G4	G5	G6	G7	SEM	*p* value
EW (g)	57.00 ± 0.55	57.92 ± 0.35	57.99 ± 0.47	66.17 ± 0.47	66.34 ± 0.50	61.97 ± 0.71	62.00 ± 0.38	0.25	< 0.001
ESW (g)	5.32 ± 0.34	5.27 ± 0.58	5.01 ± 0.61	6.50 ± 0.88	6.81 ± 0.47	5.90 ± 0.60	5.71 ± 0.49	0.35	< 0.001
ESS (kg/cm^2^)	4.49 ± 0.36	3.94 ± 0.48	3.96 ± 0.53	5.37 ± 0.27	5.72 ± 0.30	4.15 ± 0.12	4.46 ± 0.31	0.13	< 0.001
EST (mm)	0.36 ± 0.10	0.37 ± 0.02	0.37 ± 1.02	0.40 ± 0.41	0.40 ± 0.67	0.39 ± 0.21	0.36 ± 0.14	0.01	0.002
YW (g)	11.99 ± 0.76	12.17 ± 0.93	11.84 ± 0.60	12.91 ± 0.95	13.40 ± 0.81	12.84 ± 0.90	12.79 ± 0.97	0.73	0.228
AW (g)	34.76 ± 1.88	31.00 ± 1.92	33.10 ± 1.55	35.67 ± 1.03	36.78 ± 1.71	33.53 ± 1.45	35.44 ± 0.85	0.61	0.001
Haugh unit	92.24 ± 5.45	90.14 ± 4.65	90.47 ± 6.31	94.40 ± 6.06	95.25 ± 2.58	93.08 ± 5.26	93.50 ± 3.75	1.08	0.929

*Note:* Values are expressed as mean ± SEM. ESW, eggshell weight; ESS, eggshell strength; EST, eggshell thickness. Data were analyzed using one‐way ANOVA. Differences were considered statistically significant at *p* < 0.05.

Abbreviations: AW, albumen weight; EW, egg weight; YW, yolk weight.

### 3.5. Fecal Microbiota

Probiotic encapsulation supplementation had a significant influence on fecal microbial populations (Table 4). *Salmonella* sp. counts were markedly reduced in G4, G5, and G6 (5.29, 5.40, and 5.40 log_10_ CFU/g, respectively) compared with G1–G3 (7.79–8.59 log_10_ CFU/g). The lowest *Salmonella* population was recorded in G4 (5.29 log_10_ CFU/g). *E. coli* counts demonstrated moderate variation among treatments, ranging from 7.11 to 8.13 log_10_ CFU/g, with G1 showing the highest level. In contrast, lactic acid bacteria counts were elevated in probiotic‐supplemented groups, reaching 8.78 log_10_ CFU/g in G5, compared with 7.99–8.11 log_10_ CFU/g in control groups. However, no statistical difference was observed between the microbial count of E*. coli* and lactic acid bacteria.

**TABLE 4:** Effects of probiotic supplementation on fecal microbial populations (log_10_ CFU/g).

Treatments	Bacteria		
	*Salmonella* sp.	*E. coli*	Lactic acid bacteria
G1	7.79 ± 0.42^ab^	8.13 ± 0.26	8.11 ± 0.23
G2	8.00 ± 0.77^ab^	7.94 ± 0.27	8.11 ± 0.21
G3	8.59 ± 0.51^a^	8.01 ± 0.20	7.99 ± 0.45
G4	5.29 ± 0.21^c^	7.51 ± 0.23	8.44 ± 0.38
G5	5.40 ± 0.29^c^	7.41 ± 0.27	8.78 ± 0.38
G6	5.40 ± 0.19^c^	7.18 ± 0.32	8.18 ± 0.19
G7	6.19 ± 0.61^bc^	7.11 ± 0.48	8.10 ± 0.32

*Note:* Values are expressed as mean ± SEM. Data are presented as log_10_ CFU/g of fresh feces. Means within the same column with different superscript letters differ significantly (*p* < 0.05).

## 4. Discussion

This study demonstrates that a multistrain *Bacillus* formulation encapsulated with AP provides selective advantages in probiotic delivery for laying hens, particularly under thermal processing, prolonged storage, and in vivo conditions. Rather than uniformly enhancing resistance across all stressors, the benefits of GFP incorporation were most evident in heat tolerance, storage stability, and productive performance, while acid tolerance and lysozyme resistance were comparable between AO and AP formulations. *B. aryabhattai* CKNJh11 and *Bacillus* sp. THPS1 showed strong adhesion and gut tolerance, while *B. marisflavi* (OYNH19) and *Lysinibacillus* sp. PWR01 exhibited high heat and enzyme resistances. Combining them creates a consortium that can withstand various stressors from feed processing to the GIT, potentially colonizing different niches within the gut for a broader beneficial effect, as suggested by the improved performance over single‐strain or unencapsulated spores [33, 34]. In the present work, the consortium approach likely contributed to consistent performance across treatments, although the data do not support strong strain‐specific dominance under acidic or enzymatic challenges.

The high sporulation efficiency (> 96%) observed for all isolates is consistent with previous studies showing that *Bacillus* species can produce spores at high rates under optimal conditions [35, 36]. Sporulation is triggered by nutrient depletion and environmental stress, and the DSM medium used in this study provided appropriate conditions for spore formation. The spore‐forming capability is crucial for probiotic applications as spores can withstand harsh processing conditions and maintain viability during storage [37, 38]. Encapsulation further enhanced overall survivability; however, our results indicated that differences between AO and AP were not statistically significant for acid tolerance (pH 2.0–3.0) or lysozyme exposure, suggesting that AO already provides sufficient protection under these specific gastrointestinal stress conditions. The tolerance to bile salts (0.1%–0.5%) demonstrated by the encapsulated spores is essential for probiotic survival through the GIT [39, 40]. The stomach bile salt concentration in the small intestine can reach 0.3%–0.5% [41]. The encapsulation significantly enhanced SR, with the AP formulation providing high protection compared to AO. This enhanced protection may be attributed to the additional barrier properties and matrix strength provided by the GFP [42].

Heat tolerance is critical for probiotics intended for use in animal feed, as feed pelleting processes typically involve temperatures of 70°C–95°C [43, 44]. The encapsulated spores maintained > 90% viability at these temperatures, demonstrating their suitability for incorporation into commercial feed production [45, 46]. The high performance of alginate and polysaccharide beads at higher temperatures (80°C–95°C) suggests improved thermal protection compared to AO. In addition, the storage stability results are particularly significant for commercial applications [47, 48]. The ability of alginate and polysaccharide‐encapsulated spores to maintain > 88% viability after 60 days at various temperatures, including room temperature (25°C), indicates excellent shelf stability. This is substantially better than many commercial probiotic products that require refrigeration. The GFP appears to enhance the structural integrity and protective properties of the alginate matrix, preventing moisture loss and maintaining bead stability [49].

The significantly enhanced in‐growth performance of hen in the AP beads is the cornerstone of this research. The GFP likely acts as a prebiotic and a structural enhancer [21, 22, 50]. It may form a more complex and robust gel network with sodium alginate, providing a superior physical barrier against heat, acid, and enzymes. The higher spore release rate in AP beads is critical; it suggests that while the capsule is strong enough for protection, it disintegrates effectively in the gut to release the viable spores, a common challenge with some encapsulation methods [51, 52]. This finding aligns with studies showing that composite matrices can improve the functional properties of probiotic microcapsules [53–56]. Importantly, inclusion of free‐spore groups (G6 and G7 groups) enabled differentiation between intrinsic probiotic effects and matrix‐dependent delivery optimization. Although G6 (300 mg/kg) showed improvement across several productive performance indicators relative to control, the consistently superior results observed in the G5 group (AP encapsulation at 100 mg/kg) demonstrate that elevated probiotic dosage alone does not account for the enhanced production results recorded. The data suggest, rather, that the structural protection and delivery efficiency afforded by the composite matrix were critical to a decisive role in maintaining viable spore availability under gastrointestinal transit. Corroborating this interpretation, the AP outperformed both equivalent‐dose AO and higher‐dose free spores supports the hypothesis that encapsulation performance, rather than simple dose escalation, determines biological efficacy in laying hens.

The improvements in egg production performance and egg quality observed in this study align with previous research demonstrating the benefits of *Bacillus* probiotics in laying hens [14, 57–59]. The 4.2% increase in the egg production rate and significant improvement in egg mass with AP‐encapsulated spores (10^6^ CFU/day) represent economically meaningful improvements for commercial production. The enhanced eggshell quality, evidenced by increased ESW and strength, is particularly valuable as it reduces egg breakage during handling and transportation. The animal trial clearly demonstrates the practical benefit of the encapsulation strategy. The fact that the AP group showed the most significant improvement in egg production and eggshell quality, even compared to the group receiving a higher dose of free spores (600 mg probiotic spore/kg chicken feed), underscores the importance of ensuring viable probiotic delivery to the gut [54, 60, 61]. The enhanced gut health, evidenced by the reduction in pathogens (*Salmonella* sp.), directly explains the improved production metrics [62–64]. A healthier gut environment leads to better nutrient absorption and overall bird health, translating into higher productivity [65].

The mechanism by which probiotics improve production performance likely involves multiple pathways. First, probiotics can enhance nutrient digestibility by producing enzymes such as amylase, protease, and lipase, improving feed utilization [66–68]. Second, probiotics compete with pathogenic bacteria for nutrients and adhesion sites in the intestine, reducing pathogen colonization [69]. Third, probiotics modulate the immune system, enhancing both innate and adaptive immune responses [70, 71]. The significant reduction in *Salmonella* sp. populations in feces supports this multifactorial mode of action. The high efficacy of encapsulated spores compared to that of free spores may be attributed to several factors. Encapsulation provides protection during gastric transit, ensuring higher numbers of viable spores reach the intestine [72]. The controlled release properties of alginate beads may also provide sustained probiotic colonization in the gut. As discussed before, the GFP may have prebiotic effects, selectively promoting the growth of beneficial bacteria [73]. The use of a multistrain probiotic formulation rather than single strains may offer additional advantages. Different *Bacillus* species produce different antimicrobial compounds and enzymes, potentially providing broader spectrum benefits [74–76]. The combination of four *Bacillus* isolates may have synergistic effects in improving gut health and modulating the microbiota. Compared to antibiotic growth promoters, probiotics offer several advantages including no withdrawal period [6], no antibiotic residues in eggs [77–79], and reduced risk of antimicrobial resistance development [80, 81]. The results of this study demonstrate that properly formulated and delivered probiotics can achieve production benefits comparable to or exceeding those of antibiotics.

From our results, it can be seen that GFP represents an innovative encapsulation material with several advantages. As a natural, renewable resource abundant in Thai coastal waters, it offers sustainability benefits. The biocompatibility and potential prebiotic properties of seaweed polysaccharides make them particularly suitable for food and feed applications [82–84]. Future research should investigate the specific structural properties of GFP that contribute to enhanced encapsulation performance. The reduction in pathogenic bacteria without the use of antibiotics positions this AP‐encapsulated multistrain *Bacillus* probiotic as a viable alternative to antibiotic growth promoters. Its enhanced stability reduces the required dosage and simplifies storage conditions, making it more practical for industrial feed production. Additionally, the survival of multi‐*Bacillus* spores under simulated GIT conditions was compared between encapsulated and nonencapsulated forms, showing a significant increase in the encapsulated group’s SR. Consequently, our results clearly demonstrated that encapsulation with AP substantially enhanced the SR of probiotic spores when subjected to acidic pH, bile salts, and digestive enzymes, as compared to free spores. Notably, encapsulated spores exhibited higher viability across the tested GIT‐mimicking environment and time point, indicating superior protection against harsh digestive conditions. These findings support the conclusion that microencapsulation is an effective strategy to improve the stability and delivery of probiotics through the GIT, thereby increasing their potential functional benefits in animal production systems. The novelty of this work lies in demonstrating that a locally sourced marine polysaccharide can be strategically integrated with alginate to address specific industrial bottlenecks that are heat processing, nonrefrigerated storage, and in vivo productivity in laying hens. Previous studies have largely focused on single‐strain probiotics, short‐term in vitro assays, or nonpoultry models. By linking encapsulation performance with biologically and economically meaningful outcomes (egg mass and production rate), this study advances the field toward application‐oriented probiotic design. However, some limitations of this study should be acknowledged. While the 8‐week trial duration offers valuable short‐term data, longer‐term studies are necessary to evaluate the sustained effects of probiotics over complete laying cycles. Additionally, mechanistic studies (e.g., gene expression, immune markers, and detailed microbiome analysis) would provide deeper insights into the mode of action of probiotics in the gut microbiota of laying hens.

## 5. Conclusion

The coencapsulation of a multistrain *Bacillus* consortium with sodium alginate and GFP results in a highly effective and stable probiotic supplement. This system surpasses alginate‐alone encapsulation and unencapsulated spores by providing high protection during processing, storage, and gastrointestinal transit. Consequently, it ensures the maximum delivery of viable probiotics to the hen’s gut. The improvements in laying performance, egg quality, and gut health demonstrate the great potential of this technology for sustainable poultry production.

## Author Contributions

Waraphorn Sihamok: roles/writing–original draft, formal analysis, data curation, methodology, investigation, and writing–review and editing. Orathai Dangsawat: roles/writing–original draft, formal analysis, data curation, methodology, investigation, and writing–review and editing. Apinan Nuisiri: roles/writing–original draft, investigation, and methodology. Jessada Rattanawut: roles/writing–original draft, investigation, methodology, and supervision. Rapeewan Sowanpreecha: investigation, methodology, and supervision. Umaporn Pastsart: review and editing, investigation, and supervision. Chatchawan Chotimarkorn: review and editing, methodology, and conceptualization. Papungkorn Sangsawad: conceptualization, roles/writing–original draft, investigation, methodology, and supervision. Luu Tang Phuc Khang: roles/writing–original draft, writing–review and editing, investigation, methodology, and conceptualization. Orranee Srinual: conceptualization, roles/writing–original draft, writing–review and editing, investigation, and methodology. Nguyen Vu Linh: conceptualization, roles/writing–original draft, writing–review and editing, investigation, and methodology. Patima Permpoonpattana: roles/writing–original draft, formal analysis, data curation, methodology, investigation, writing–review and editing, resources, conceptualization, funding acquisition, and project administration.

## Funding

This research was supported by National Science, Research and Innovation Fund (NSRF) and Prince of Songkla University (Grant No. IAF6801367S).

## Conflicts of Interest

The authors declare no conflicts of interest.

## Data Availability

The datasets generated and analyzed during this study are available from the corresponding authors upon reasonable request.
